# Chronic Total Occlusions in Sweden – A Report from the Swedish Coronary Angiography and Angioplasty Registry (SCAAR)

**DOI:** 10.1371/journal.pone.0103850

**Published:** 2014-08-12

**Authors:** Truls Råmunddal, Loes Hoebers, Jose P. S. Henriques, Christian Dworeck, Oskar Angerås, Jacob Odenstedt, Dan Ioanes, Göran Olivecrona, Jan Harnek, Ulf Jensen, Mikael Aasa, Risto Jussila, Stefan James, Bo Lagerqvist, Göran Matejka, Per Albertsson, Elmir Omerovic

**Affiliations:** 1 Department of Cardiology, Sahlgrenska University Hospital, Gothenburg, Sweden; 2 Department of Cardiology, Academic Medical Center, Amsterdam, The Netherlands; 3 Department of Coronary Heart Disease, Skåne University Hospital, Scania, Sweden; 4 Department of Cardiology, Stockholm South General Hospital, Stockholm, Sweden; 5 Department of Medical Sciences, Uppsala University, Uppsala, Sweden; University Hospital Medical Centre, Germany

## Abstract

**Introduction:**

Evidence for the current guidelines for the treatment of patients with chronic total occlusions (CTO) in coronary arteries is limited. In this study we identified all CTO patients registered in the Swedish Coronary Angiography and Angioplasty Registry (SCAAR) and studied the prevalence, patient characteristics and treatment decisions for CTO in Sweden.

**Methods and Results:**

Between January 2005 and January 2012, 276,931 procedures (coronary angiography or percutaneous coronary intervention) were performed in 215,836 patients registered in SCAAR. We identified all patients who had 100% luminal diameter stenosis known or assumed to be ≥3 months old. After exclusion of patients with previous coronary artery bypass graft (CABG) surgery or coronary occlusions due to acute coronary syndrome, we identified 16,818 CTO patients. A CTO was present in 10.9% of all coronary angiographies and in 16.0% of patients with coronary artery disease. The majority of CTO patients were treated conservatively and PCI of CTO accounted for only 5.8% of all PCI procedures. CTO patients with diabetes and multivessel disease were more likely to be referred to CABG.

**Conclusion:**

CTO is a common finding in Swedish patients undergoing coronary angiography but the number of CTO procedures in Sweden is low. Patients with CTO are a high-risk subgroup of patients with coronary artery disease. SCAAR has the largest register of CTO patients and therefore may be valuable for studies of clinical importance of CTO and optimal treatment for CTO patients.

## Introduction

Chronic total occlusions (CTO) are difficult to treat with percutaneous coronary intervention (PCI) [1]. Revascularization of CTO demands expert skills, longer procedural time and is associated with higher procedural risks such as coronary perforation, contrast nephropathy, radiation exposure, and loss of collateral circulation [1], [2]. According to the European and American guidelines, PCI of CTO has class-IIa recommendation (weight of evidence in favour of the treatments usefulness/efficacy) [3], [4] but this recommendation is based on small retrospective studies and on expert consensus.

The true prevalence of a CTO in the general population is unknown and few studies have addressed this question. In observational studies, a CTO was found in approximately one third of the patients referred for coronary angiography [5]–[7]. However, these studies were based on low number of patients and participating hospitals, and may therefore be prone to selection bias. To date, no epidemiological study has investigated the prevalence and clinical characteristics of CTO patients at the nationwide level.

The Swedish Coronary Angiography and Angioplasty Registry (SCAAR) is a prospective national registry that collects data about all patient undergoing coronary angiography and PCI in Sweden [8]. Therefore, we identified all CTO patients registered in SCAAR and studied prevalence, patient characteristics and treatment decision for CTO in Sweden.

## Methods

### Swedish Coronary Angiography and Angioplasty Registry (SCAAR)

The SCAAR registry was established in 1999 after the unification of Swedish Coronary Angiography registry (Acta Coronaria) and the Swedish Coronary Angioplasty registry (SCAP). SCAAR, which is a part of the national SWEDEHEART registry, holds data on all consecutive patients from all centres that perform coronary angiography and PCI in Sweden. The registry is independent of commercial funding and is sponsored by the Swedish Health Authorities only. The technology has been developed and administered by the Uppsala Clinical Research Center. Since 2001, SCAAR has been Internet-based, with recording of data online through an Internet interface in the catheterization laboratory; data are transferred in an encrypted format to a central server at the Uppsala Clinical Research Center.

In total, there are 30 hospitals with cardiac catheterization facilities in Sweden of which 9 are university hospitals. In SCAAR, a coronary angiography procedure is described by approximately 50 variables while a PCI procedure allows for is described by approximately 200 variables. The information about clinical characteristics and procedural details is entered into the registry immediately after the procedure by the PCI physician after the review of clinical information.

### Ethics statement

The study was approved by the regional ethical review board of Gothenburg University, Gothenburg,Sweden. The regional ethical review board waived the need for written informed consent from the participants according to Swedish legislation and because data were de-identified and anonymized before analysis.

### Definitions

We defined CTO as 100% luminal diameter stenosis and the absence of antegrade flow known or assumed to be ≥3 months duration [6], [9], [10]. Coronary artery disease was defined as a luminal narrowing ≥50% on angiography. Procedural success after PCI treatment of the coronary lesion is defined as residual stenosis <50%, decreased grade of stenosis after intervention by at least 20%, normal blood flow and no serious complications.

### Study cohort

We used two different methods to identify CTO patients in SCAAR between January 2005 and January 2012.

The first method is based on the information about %-luminal stenosis at the level of coronary segments that was introduced in 2005. From this date onwards, the information derived from a diagnostic coronary angiogram can also be used to determine if a coronary segment was totally occluded. In order to differentiate between acute and chronic occlusions, we excluded patients who underwent a procedure for acute coronary syndrome in whom the occlusion was located in the same coronary artery as the culprit vessel. Furthermore, we excluded patients who underwent a procedure in the same vessel within the previous 3 months. The CTO patients identified by this method constitute the *coronary segment subcohort*.

The second method is based on the separate variable by which PCI operators classify a treated occlusion either as a chronic occlusion ≥3 months duration or as an acute/subacute occlusion ≤3 months duration.

The patients with previous coronary artery bypass graft (CABG) surgery were excluded from analysis as the patency of the graft could not be determined. The study was scrutinised and approved by the ethics board according to the Swedish law and regulations.

### Total CTO cohort

**Figure 1:**
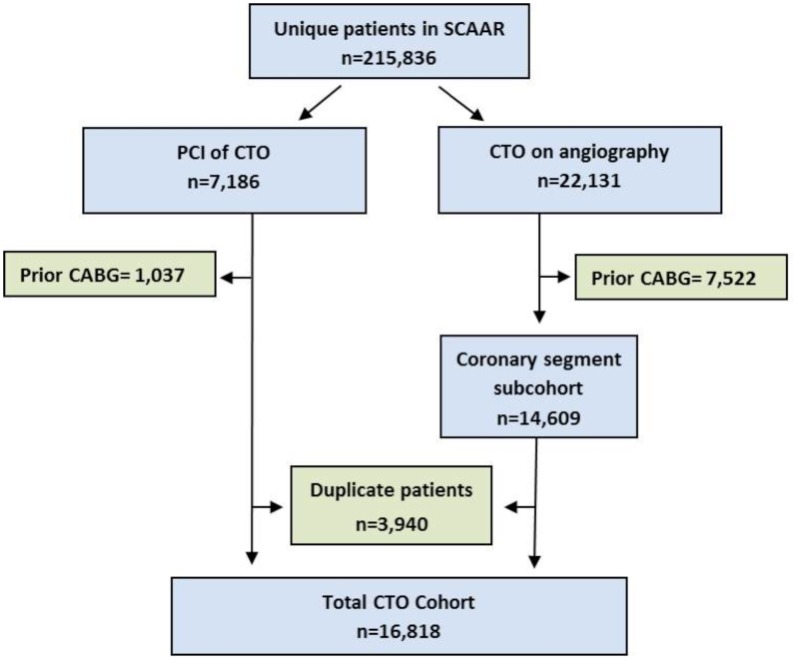
Flow chart for identification and selection of CTO patients in SCAAR. Based on the selection methods we have defined two CTO groups. The first group - *the total CTO cohort-* contains all CTO patients recognized by one or both methods during the period. The second group is the subcohort that contains the patients in whom a CTO was identified through the %-luminal stenosis on the coronary segments – *the coronary segment subcohort*.

The total CTO cohort contains all CTO patients identified by either of the two methods (**Figure 1**). Patients and procedures in which the same CTO lesion was registered through both methods or at multiple occasions were identified and duplicate observations were excluded from the analysis. For each patient, the procedure where the CTO was observed first was selected.

### Coronary segment subcohort

We also present data from the subcohort of patients who were identified according to whether they had a 100% luminal stenosis and considered to be a CTO based on the above mentioned inclusion criteria. We compared patient characteristics and treatment decisions between patients with significant coronary artery disease in whom a CTO was observed or not. Prevalence of a CTO was calculated only from the coronary segment subcohort and in relation to three different denominators: the number of unique procedures, the number of unique patients and the number of unique patients with significant coronary artery disease.

### Validation of CTO

Validation of the CTO definition was performed in a subgroup of 955 patients from one university hospital (Sahlgrenska University Hospital) and from three county hospitals (Norra Älvsborgs Hospital, Borås Hospital, Skövde Hospital). This subgroup represents 5.7% of all identified CTO patients in SCAAR in the study period. The patients were randomly selected by means of random number generator using Stata software (Version 12.1, StataCorp, College Station, Texas, USA). The validation procedure was conducted by a panel consisting of five experienced interventional cardiologists. The panellists examined individual coronary angiograms according to a monitoring plan defined in advance. Each angiogram was evaluated in regard to whether the patient had previous CABG, whether the treated occlusion was ≥3 months old and whether 100% segmental stenosis on angiogram was an occlusion ≥3 months old. The results from the validation procedure were then compared to the data entered in SCAAR.

### Statistical analysis

Differences in baseline characteristics between the groups were tested by the χ^2^ test for categorical variables while Mann–Whitney U test and Kruskal-Wallis test were used for comparison of continuous non-normally distributed variables. We used Shapiro-Wilks test to test for normal distribution. Tests for trends were made using linear contrasts of means in a one-way analysis of variance model for numerical data and the Armitage-Cohrane trend test for categorical data. We used logistic regression with test for linear trend to evaluate whether annual incidence of CTO and success rate for PCI of CTO changed during the study period. Statistical significance was defined as a P-value<0.05. All analyses were performed using Stata software (Version 13.1, StataCorp, College Station, Texas, USA).

## Results

### Prevalence of CTO in Sweden

**Figure 2:**
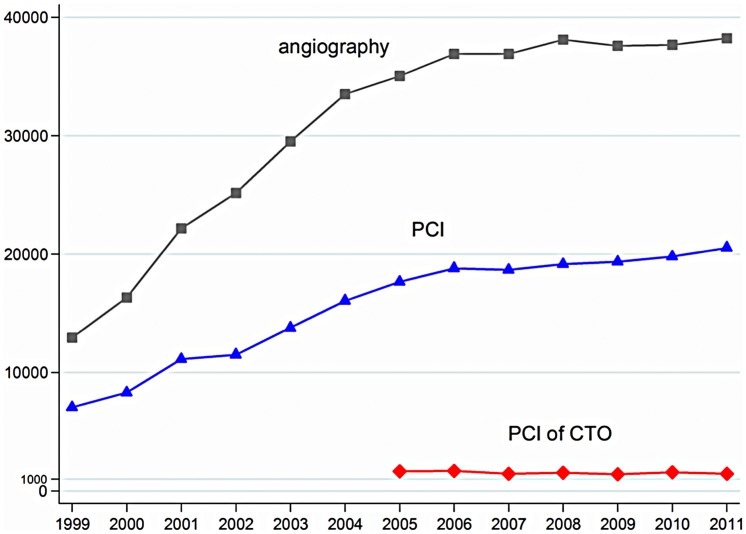
Annual number of coronary angiographies, PCI's, and PCI's performed in CTO patients in Sweden reported in SCAAR since 1999.

As of January 2012, 497,572 procedures (coronary angiographies and PCIs) performed in 348,863 patients were registered in SCAAR. The numbers of PCIs and coronary angiographies increased since 1999 (**Figure 2**). The annual rate of PCIs for CTO remained low (∼1200 in 2011).

Between January 2005 and January 2012, Swedish interventionalists completed 276,931 procedures in 215,836 patients. The total number of CTO patients without previous CABG during the same period was 16,818 and these patients constitute the total CTO cohort (**Figure 1**).

Information about age of the treated CTO was missing in 0.8% (n = 1077) of all procedures. Of the 134,087 reported PCIs, 7,816 (5.8%) involved the treatment of a CTO. These procedures were performed in 29 different hospitals on 7,186 unique patients of whom 6,149 without prior CABG. Almost half (43%) of all CTO procedures were performed at university hospitals. Data on procedural success was missing in 12 procedures (0.2%). The overall success rate was 53.1%. The annual success rate did not change significantly during the study period with 54.4% in 2005 and 56.6% in 2012 (OR 1.02, 95% CI 0.99–1.06; P = 0.15 test for linear trend).

Complete information about luminal %-stenosis was available in 160,159 (57.8%) angiographies of which 144,744 were from in patients without previous CABG. A CTO was observed in 10.6% of angiographies from 126,745 patients. Of these patients, 14,609 had at least one CTO resulting in a prevalence of 11.5%. Coronary artery disease was diagnosed in 91,154 patients of which 16.0% had a CTO. In patients who underwent multiple procedures, the CTO was diagnosed on the first diagnostic angiogram in 90% of the cases. The annual number of diagnosed CTO in patients undergoing coronary angiography decreased gradually by 25% from 11.5% in 2005 to 8.6% in 2012 (OR 0.97; 95% CI 0.96–0.98; P<0.001 test for linear trend). In patients with significant coronary artery disease, CTO decreased by 12% from 17.2 in 2005 to 15.1 in 2012 (OR 0.95; 95% CI 0.94–0.96; P<0.001 test for linear trend).

### Patient characteristics

The clinical characteristics of all CTO patients - the total CTO cohort - registered in SCAAR since 2005 are summarized in **Table 1**. The majority of CTO patients were male. The high occurrence of previous MI (37%) and the presence of traditional cardiovascular risk factors make CTO patients a high-risk population.

**Table 1:** Baseline characteristics of the total CTO cohort in SCAAR at the time of diagnosis based on data collected during the period 2005–2012.

	All CTO patients (n = 16,818)	Missing %
**Male** (%)	77.5	0.0
**Age** (median, IQR)	68 (60–76)	0.2
**Diabetes** (%)	23.9	1.0
**Hypertension** (%)	61.9	2.5
**Hyperlipidemia** (%)	62.7	2.9
**Smoking status** (%)		6.2
Current smoker	19.9	
Previous smoker	40.2	
**Previous MI** (%)	37.2	3.8
**Previous PCI** (%)	18.4	0.1
**Cardiogenic shock** (%)*	10.2	1.2
**Creatinine Clearance** (ml/min)	81 (61–104)	28.2
**CCS class** (%)**		7.0
I	9.0	
II	52.2	
III	37.4	
IV	1.4	
**Indication** (%)		0.0
Stable CAD	45.5	
Unstable CAD/NSTEMI	27.5	
STEMI	14.3	
Other	12.7	
**Extent of CAD** (%)		1.1
1 vessel	20.4	
2 vessel	35.1	
3 vessel	36.1	
Left main disease***	8.4	

CAD: coronary artery disease, CCS: Canadian cardiovascular society, CTO: chronic total occlusion, IQR: inter quartile range, MI: myocardial infarction, (N)STEMI: (non-)ST-elevation myocardial infarction, PCI: percutaneous coronary intervention.

* Cardiogenic shock only displayed for the indication STEMI.

**CCS class only displayed for the indication stable CAD.

*** Left main (LM) disease includes: LM+1 vessel, LM+2 vessel and LM+3 vessel.

**Figure 3:**
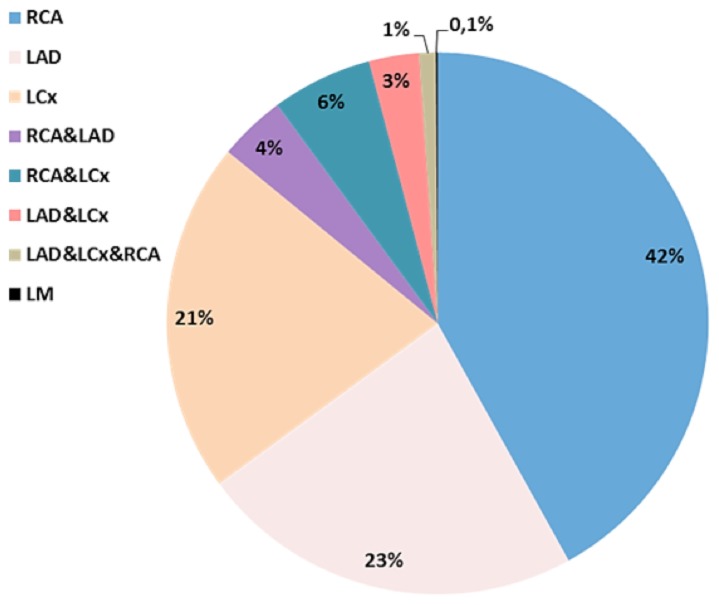
Coronary location of CTO observed at angiography. RCA = right coronary artery, LAD = left descending coronary artery, LCx = left circumflex coronary artery, LM = left main.

**Table 2** shows the baseline demographic and angiographic characteristics of patients with coronary artery disease stratified for the presence of a CTO on angiography (coronary segment subcohort). CTO patients were more often males and were more likely to have risk factors including previous MI. In addition, the extent of coronary artery disease was more severe in CTO patients with more multivessel and left main disease. Although CTO was diagnosed in the majority of cases during a coronary angiography for stable angina, a substantial proportion was diagnosed in patients with acute coronary syndrome. Furthermore, patients with a CTO more often presented in cardiogenic shock at presentation for STEMI compared to patients without a CTO. The CTO was more frequently located in the right coronary artery (RCA) followed by the left anterior descending artery (LAD) and left circumflex artery (LCx) (**Figure 3**). In approximately 14%, CTOs were observed in more than one vessel of which only 0.1% included the left main.

**Table 2:** Baseline characteristics of patients from coronary segment subcohort with coronary artery disease, stratified for the presence of a CTO observed on angiography.

	CTO observed (n = 14,609)	Missing %	CTO not observed (n = 76,545)	Missing %	P-value
**Male gender** (%)	77.7	0	70.6	0.0	<0.01
**Age** (median, IQR)	68 (61–76)	0.2	68 (60–75)	0.2	<0.01
**Diabetes** (%)	24.0	1.0	18.6	0.8	<0.01
**Hypertension** (%)	62.0	2.4	52.5	2.0	<0.01
**Hyperlipidemia** (%)	61.9	2.8	44.4	2.5	<0.01
**Smoking status** (%)		5.8		5.5	<0.01
Current smoker	20.0		21.6		
Previous smoker	40.0		35.0		
**Previous MI** (%)	37.0	3.8	16.5	2.3	<0.01
**Previous PCI** (%)	17.5	0.1	10.7	0.05	<0.01
**Cardiogenic shock**(%)*	10.1	1.0	3.5	1.3	<0.01
**Creatinine Clearance**	80	28.2	81	34.2	<0.01
(ml/min)	(60–103)		(62–103)		
**CCS class** (%)**		6.9		5.5	0.19
I	9.3		9.6		
II	53.4		54.7		
III	36.0		34.5		
IV	1.3		1.2		
**Indication** (%)		0		0	<0.01
Stable CAD	44.5		17.1		
Unstable CAD/NSTEMI	26.3		49.6		
STEMI	15.1		26.0		
Other	14.0		7.3		
**Extent of CAD** (%)		1.5		1.2	<0.01
1 vessel	17.4		48.7		
2 vessel	35.1		25.9		
3 vessel	38.3		17.2		
Left main disease***	9.2		8.2		

CAD: coronary artery disease, CCS: Canadian cardiovascular society, CTO: chronic total occlusion, IQR: inter quartile range, MI: myocardial infarction, (N)STEMI: (non-)ST-elevation myocardial infarction, PCI: percutaneous coronary intervention.

* Cardiogenic shock only displayed for the indication STEMI.

**CCS class only displayed for the indication stable CAD.

*** Left main (LM) disease includes: LM+1 vessel, LM+2 vessel and LM+3 vessel.

### Treatment of CTO patients

**Table 3** shows the differences in baseline demographic and angiographic characteristics of CTO patients identified through the coronary segment stenosis, according to the received treatment at baseline. After diagnosis, the majority of the CTO patients (56%) received medical treatment only. The CTO patients who received invasive treatment were evenly distributed between PCI (22.3%) and referral for CABG (21.7%). CTO patients, who received medical treatment only, had more often previous MI, presented more often with STEMI, had a lower creatinine clearance and had less severe angina symptoms in comparison to the patients who were treated invasively for a CTO. Patients who were referred for CABG were more often male with a higher prevalence of cardiovascular risk factors including diabetes, had less often previous MI or PCI, presented more often with stable angina and suffered more frequently from extensive coronary artery disease than CTO patients treated with PCI. CTO patients treated for stable angina received medical treatment in 41.6%, PCI in 20.6% and CABG in 37.8%. CTO patients who were treated for acute coronary syndrome received medical treatment in 28.6%, PCI in 71.2%, and were referred to CABG in 0.2%. Procedure-related complications were reported in 5.4% CTO patients treated with PCI. The following complications were reported: death (0%), major bleeding (1.3%), stroke (0.3%), pericardial tamponade (0.4%) renal insufficiency (0.7%), emergency PCI (0.1%), emergency CABG (0%), procedure-related MI (1.1%), anaphylactic reaction (0.1%), other (1.4%)

**Table 3:** Baseline characteristics of CTO patients from coronary segment subcohort according to the treatment strategy.

	No PCI of CTO (n = 8,182)		PCI of CTO (n = 3,251)		Referral for CABG (n = 3,172)		
	(%)	Missing (%)	(%)	Missing (%)	(%)	Missing (%)	P-value
**Male gender**	76.2	0	76.5	0	82.9	0	<0.01
**Age** (median, IQR)	69 (61–77)	0.2	66 (59–74)	0.2	68 (61–74)	0.2	<0.01
**Diabetes**	24.0	1.1	21.0	1.2	26.9	0.4	<0.01
**Hypertension**	61.6	3.0	59.8	2.3	65.2	1.5	<0.01
**Hyperlipidemia**	58.6	3.4	60.2	2.7	71.2	1.3	<0.01
**Smoking status**		7.0		5.0		3.6	<0.01
Current smoker	21.4		20.7		15.5		
Previous smoker	39.4		39.5		42.1		
**Previous MI**	41.3	4.1	33.4	3.5	29.4	3.2	<0.01
**Previous PCI**	19.9	0.1	20.3	0.2	8.3	0.03	<0.01
**Cardiogenic Shock** **	10.0	1.0	10.4	0.9	0	0	0.84
**Creatinine Clearance** (ml/min)	77 (57–101)	31.6	85 (65–109)	26.1	82 (64–102)	21.8	<0.01
**CCS class** *		7.1		4.6		7.8	<0.01
I	13.2		8.6		5.3		
II	55.2		56.3		49.8		
III	30.3		34.3		43.3		
IV	1.4		0.8		1.6		
**Indication**		0		0		0	<0.01
Stable CAD	33.1		41.3		77.4		
Unstable CAD/NSTEMI CAD/NSTEMI	31.1		39.8		0.4		
STEMI	21.7		13.5		0		
Other	14.2		5.5		22.2		
**Extent of CAD (%)**		1.2		1.3		1.3	<0.01
1 vessel	14.9		36.5		4.2		
2 vessel	40.9		39.6		15.8		
3 vessel	37.9		21.1		56.8		
Left main disease***	6.3		2.8		23.2		

CAD: coronary artery disease, CCS: Canadian cardiovascular society, CTO: chronic total occlusion, IQR: inter quartile range, MI: myocardial infarction, (N)STEMI: (non-)ST-elevation myocardial infarction, PCI: percutaneous coronary intervention.

* Cardiogenic shock only displayed for the indication STEMI.

**CCS class only displayed for the indication stable CAD.

*** Left main (LM) disease includes: LM+1 vessel, LM+2 vessel and LM+3 vessel.

Data about initial treatment strategy were missing in four CTO patients.

### Validation of CTO

The validation analysis revealed 36 (3.8%) erroneously classified patients. Of these, 18 patients did not have a 100% occluded coronary artery on the coronary angiogram. Another 5 patients had prior CABG and 13 patients had acute or subacute coronary occlusions.

## Discussion

In this study, we identified and studied 16,818 CTO patients in SCAAR. We found CTO in every tenth patient undergoing coronary angiography, and that the prevalence of CTO in Sweden decreased by one quarter in these patients between January 2005 and January 2012.

The true prevalence of a CTO in the general population is unknown and not well studied. In a few older studies based on small populations, CTO prevalence was 35% [7] and 52% [5] in patients with significant coronary artery disease. In a recent study from Canada based on 1697 patients, CTO prevalence was 14.7% in all patients undergoing angiography [6]. However, our study shows that the prevalence of CTO was 11.5% in Sweden. The reason for the difference in CTO prevalence between Sweden and other countries may be due to selection bias. While all previous studies were based on selected and relatively small populations, SCAAR holds information from all hospitals that perform coronary angiography and PCI in Sweden which reduces selection bias. Another explanation may be that prevalence of CTO differs between the countries due to variance in severity of coronary artery disease, treatment algorithms for acute coronary syndromes and organization of health care system [11].

Our study shows that CTO patients are a high-risk population with more traditional cardiovascular risk factors, multivessel disease, history of MI and PCI, which is in accordance with previous reports [1], [6], [9]. Furthermore, a substantial number (14%) of patients had multiple CTO's in separate vessels.

Majority of CTO patients had stable angina but approximately 40% had acute coronary syndrome at the time when CTO was diagnosed. Overall, 56% of the CTO patients were treated with medical treatment initially. The remaining patients were evenly distributed between percutaneous and surgical revascularization similar to the Canadian study [9]. The success rate for CTO procedures in all Swedish hospitals was 53% which is lower than 70% and 80% reported by others [2], [6], [12]. However, the success rate in this study is based on all CTO procedures performed in our country rather than in a single hospital or smaller registries in other studies. The average success of 53% in SCAAR is unlikely to be representative for low- versus high-volume CTO centers [2], [10]. Among the 30 hospitals reporting to SCAAR there are only a few high-volume centres with a dedicated CTO program and less than half of the CTO procedures in Sweden were performed at university hospitals. Some evidence suggest that procedural success is closely related to operator experience [13]. However, the recommendation that CTO procedures should be concentrated to dedicated high-volume hospitals needs stronger evidence.

Although CTO patients are common in clinical work, the evidence for the current guidelines and clinical practice is limited. The need for randomized clinical trials in this patient population is pressing; however, only a few such trials are initiated and on-going [12], [14]. Until evidence from randomized trials becomes available we will need to identify and utilize the information available from contemporary databases and quality registries. The SCAAR registry with its distinctive structure covering the whole Swedish nation provides unique possibility to study several important features of CTO's including epidemiology, patients characteristic as well as health outcomes. It contains information about both past and current treatment strategies of these complex patients not only from specialized centers, but also from all hospitals that preforms PCI. Given these circumstances, the SCAAR registry can be an important instrument to address many key questions in CTO.

The prevalence of CTO in Sweden decreased by one quarter during the study period. We hypothesize that this primarily reflects the increasing rate of timely revascularisation with PCI of patients with acute coronary syndrome – STEMI and non-STEMI. Because the prevalence decreased by 25% while the number of procedures remained unchanged, the proportion of CTO procedures increased by the same percentage. Besides the decreased prevalence, the relatively low annual rate of CTO procedures in Sweden may be related to improved treatment of symptoms, better quality of life, fear of complications, technical complexity, and low evidence-level.

There are some important limitations that need to be addressed. First, this is an observational study and as such it provides only associative evidence, not causative. Second, we cannot rule out the possibility of selection bias, as only hospitalized patients are included in the registry. Substantial proportion of missing data in the coronary segment subcohort may have resulted in biased estimate on CTO prevalence. Third, patients with missing data tend to have higher risk and their exclusion from the analysis might have produced biased results. Fourth, we cannot exclude the possibility that some occlusions had duration of less than three months, however the validation of CTO diagnosis and procedures in SCAAR have shown that only 3.8% were erroneously classified.

## Conclusions

SCAAR is the largest database of CTO patients to date. CTO is a frequent finding in Swedish population and is diagnosed in every 10^th^ patient undergoing coronary angiography. The prevalence of CTO has decreased by one quarter. Patients with a CTO represent a high risk subgroup of CAD patients. SCAAR may be a valuable source of data in the process of evidence-building in the CTO field.
