# Correction: Chronic Total Occlusions in Sweden – A Report from the Swedish Coronary Angiography and Angioplasty Registry (SCAAR)

**DOI:** 10.1371/journal.pone.0112370

**Published:** 2014-10-27

**Authors:** The PLOS ONE Staff

The second author’s name is spelled incorrectly. The correct name is Loes P. Hoebers. The correct citation is: Råmunddal T, Hoebers LP, Henriques JPS, Dworeck C, Angerås O, et al. (2014) Chronic Total Occlusions in Sweden – A Report from the Swedish Coronary Angiography and Angioplasty Registry (SCAAR). PLoS ONE 9(8): e103850. doi:10.1371/journal.pone.0103850
